# The role of antenatal education on maternal self-efficacy, fear of childbirth, and birth outcomes: A systematic review and meta-analysis

**DOI:** 10.18332/ejm/200747

**Published:** 2025-03-04

**Authors:** Amal Zaman, Hammad A. Fadlalmola, Sara E. Ibrahem, Fathia H. Ismail, Huda H. Abedelwahed, Amira M. Ali, Nafesa H. Abdelgadim, Amna M. A. Mustafa, Insaf H. Ahmed, Nasreldeen M. Ahmed, Amna A. Eltyeb, Dalia A. Gaafar, Soad M. Alnassry, Afaf A. Adam, Nagat S. Yasin, Rasha A. Ali, Aida A. Fadlalla, Amira E. Eltayeb, Amira M. Saad

**Affiliations:** 1 College of Medicine, Department of Obstetrics and Gynecology, Taibah University, Al Madinah Munawara, Saudi Arabia; 2 Department of Community Health Nursing, Nursing College, Taibah University, Al Madinah Munawara, Saudi Arabia; 3 Department of Nursing, College of Nursing and Health Sciences, Jazan University, Jazan, Saudi Arabia; 4 Department of Nursing, College of Applied Medical Sciences, Buraydah Private Colleges, Qassim, Saudi Arabia; 5 Department of Psychiatric Nursing and Mental Health, Faculty of Nursing, Alexandria University, Smouha, Alexandria, Egypt; 6 Department of Human Physiology, Jazan University, Saudi Arabia; 7 Nursing College, Al Baha University, Al Baha, Saudi Arabia; 8 College of Applied Medical Sciences, University of Hafr Al Batin, Saudi Arabia; 9 Faculty of Nursing, Tanta University, Egypt

**Keywords:** antenatal education, maternal self-efficacy, fear of childbirth, birth outcomes, systematic review

## Abstract

**INTRODUCTION:**

Antenatal education programs aim to prepare expectant mothers for childbirth and early parenthood. This meta-analysis assessed the impact of these programs on maternal psychological outcomes and birth experiences, focusing on childbirth self-efficacy, fear of childbirth, and maternal and neonatal outcomes, including rates of vaginal delivery, cesarean section, Apgar scores, and birth weight.

**METHODS:**

A systematic search was conducted in PubMed, Web of Science, SCOPUS, and Cochrane Library until July 2024. Randomized controlled trials (RCTs) comparing antenatal education to standard care were included. Data were synthesized using meta-analysis with standardized mean differences (SMD) for continuous outcomes and risk ratios (RR) for dichotomous outcomes.

**RESULTS:**

Forty studies were reviewed, with 31 eligible for meta-analysis. Among 1116 pregnant women, antenatal education significantly increased childbirth self-efficacy (SMD=2.00; 95% CI: 1.06–2.95, p<0.0001) and decreased fear of childbirth (SMD= -1.26; 95% CI: -1.79 – -0.74, p<0.00001). Maternal outcomes showed higher vaginal delivery rates (RR=1.10; 95% CI: 1.04–1.16, p=0.0004) and lower cesarean rates (RR=0.80; 95% CI: 0.70–0.92, p=0.001). No significant differences were found in episiotomy rates, Apgar scores, or birth weight.

**CONCLUSIONS:**

Antenatal education improves maternal psychological outcomes and promotes vaginal delivery. However, high heterogeneity and potential bias in the studies limit generalizability. More research is needed on long-term impacts and effectiveness in low-resource settings.

## INTRODUCTION

Pregnancy and childbirth are transformative experiences in a woman’s life, accompanied by significant physical, emotional, and psychological changes^1,2^. As expectant mothers navigate this journey, they often face uncertainties, anxieties, and fears about the birthing process and their ability to care for a newborn^3^. In recent years, there has been growing recognition of the importance of antenatal education in preparing women for childbirth and early parenthood^4^.

Antenatal education, also known as childbirth preparation or prenatal classes, typically offers expectant parents information on pregnancy, labor, delivery, and early infant care^5^. These programs vary in content and delivery methods but aim to empower women with knowledge and skills to manage pregnancy, childbirth, and postpartum^6^. While the primary goal of antenatal education is to improve maternal and neonatal outcomes, its potential to influence psychological factors such as self-efficacy and fear of birth has gained increasing attention from researchers and healthcare professionals^7,8^.

Self-efficacy, introduced by Bandura^9^, refers to an individual’s belief in their ability to perform specific tasks or behaviors successfully. In childbirth and parenting, self-efficacy is crucial in how women approach and experience these life-changing events^10^. Higher levels of self-efficacy have been associated with improved coping mechanisms during labor, reduced pain perception, and greater satisfaction with the birthing experience^11^. Furthermore, mothers with higher self-efficacy in childcare tend to exhibit more positive parenting behaviors and adapt more readily to the challenges of early parenthood^12^.

On the other hand, fear of birth, also known as tokophobia, is a significant concern for many expectant mothers^13^. This fear can range from mild anxiety to severe phobia and may have detrimental effects on both maternal and fetal well-being^14^. Women experiencing high levels of fear of birth are more likely to request elective cesarean sections, experience prolonged labor, and report negative birth experiences^15^. Additionally, fear of birth has been linked to an increased risk of postpartum depression and difficulties in mother-infant bonding^16^.

Various sociocultural factors and healthcare system variables further complicate the relationship between antenatal education, self-efficacy, and fear of birth. Cultural beliefs and practices surrounding pregnancy and childbirth can significantly influence women’s expectations and experiences^17^. These cultural perspectives shape how women perceive their ability to cope with childbirth and their level of fear. Additionally, the structure and accessibility of healthcare systems play a crucial role in determining the quality and reach of antenatal education programs^18^. Factors such as healthcare policies, resource allocation, and the training of healthcare providers all contribute to the effectiveness of these interventions^18^. Moreover, the increasing trend toward digital health solutions has led to online antenatal education programs, offering new opportunities and challenges in preparing expectant mothers for childbirth and parenting^19^.

Antenatal education programs aim to improve maternal psychological outcomes and have significant implications for maternal and neonatal birth outcomes. These programs can influence various aspects of childbirth, including the mode of delivery, with studies showing increased rates of vaginal delivery and decreased rates of cesarean sections among participants. Additionally, antenatal education has been associated with better neonatal outcomes, such as higher Apgar scores and healthier birth weights. By equipping expectant mothers with knowledge and coping strategies, these programs can enhance maternal confidence and reduce anxiety, leading to more positive birth experiences and improved health outcomes for both mothers and their newborns.

Despite the growing body of research on antenatal education programs, there remains a significant gap in the literature regarding their comprehensive impact on both maternal psychological outcomes and birth outcomes. Previous studies have often focused on isolated aspects, such as self-efficacy or fear of childbirth, without providing a holistic view of how these programs influence a range of maternal and neonatal outcomes. Additionally, the variability in study designs and outcome measures has led to inconsistent findings, making it challenging to draw definitive conclusions. This meta-analysis aims to address these gaps by systematically evaluating the impact of antenatal education on maternal psychological outcomes, as well as its secondary aim of assessing the effects on birth outcomes, including mode of delivery, Apgar scores, and infant birth weight. By investigating these relationships, we aim to provide valuable insights that can inform the development and implementation of more effective antenatal education interventions.

## METHODS

The current study followed the Preferred Reporting Items for Systematic Reviews and Meta-Analyses (PRISMA) 2020 guidelines and the Cochrane Handbook for Systematic Reviews of Interventions^20,21^.

### Information sources and search strategy

We used generic terms to search databases like PubMed, Scopus, Web of Science, and Cochrane Library to identify relevant articles from inception to July 2024. The search was performed using relevant keywords and Medical Subject Headings terms related to antenatal education, self-efficacy, fear of birth, and maternal and neonatal outcomes. Additionally, we manually searched the references of published articles to identify trials not found in the other databases. The detailed search strategy and terms are shown in Supplementary file Table 1.

### Study selection

To minimize bias, two independent reviewers (SEI and FHI) screened the titles and abstracts of all available records after removing identifying data such as author names and affiliations. They used a checklist of eligibility criteria to guide their screening process. A third reviewer (HHA) was available to resolve any conflicts. After this initial screening, the two reviewers evaluated the full texts of the selected articles for eligibility, comparing their results to resolve any remaining disagreements.

### Eligibility criteria

Studies were eligible for inclusion if they met the following criteria: the study design had to be a randomized controlled trial (RCT); and participants were required to be pregnant women, focusing the review on antenatal education’s impact on maternal self-efficacy, fear of childbirth, and birth outcomes. Studies were excluded if they were not an RCT, did not involve pregnant participants, single-arm studies, or reviews. The incuded studies were grouped for the syntheses based on the specific outcomes they measured, such as self-efficacy, fear of childbirth, and various birth outcomes.

### Data items

The intervention of interest was antenatal education programs, comparing their effectiveness to standard care or no intervention. In this study, there were changes in self-efficacy regarding childbirth and fear of childbirth. Self-efficacy in childbirth was typically measured using validated scales such as the Childbirth Self-Efficacy Inventory^22^ or similar scales. These scales assess women’s confidence in coping with labor and delivery, with higher scores indicating increased self-efficacy and a positive outcome. Fear of childbirth was commonly evaluated using tools like the Wijma Delivery Expectancy/Experience Questionnaire^23^ or other validated fear of childbirth scales. Lower scores on these scales indicate a reduction in fear, considered a favorable outcome. Secondary outcomes assessed maternal outcomes, such as the frequency of vaginal delivery, cesarean section, and episiotomy, as well as neonatal outcomes, including Apgar scores at 1 and 5 minutes, infant birth weight, and the incidence of low birth weight. All results compatible with each outcome domain in each study were sought, including all measures, time points, and analyses if applicable. If not all results were collected, methods used to decide which results to collect were based on the relevance and availability of data. Other variables for which data were sought included participant characteristics (e.g. age, parity, socio-economic status) and intervention characteristics (e.g. type, duration, and frequency of antenatal education). Assumptions were made about missing or unclear information by contacting study authors for clarification.

### Data extraction and quality assessment

Data were extracted from the included studies using a standardized form. Extracted information included study characteristics, participant demographics, participants’ characteristics related to maternity history (e.g. gestational weeks), intervention details, outcome measures, and results. Two investigators (AAEA and NHA) independently extracted the data, and a third investigator (DAG) assisted in resolving disagreements.

The methodological quality of the included studies was assessed using the Cochrane risk of bias tool for randomized trials version 2 (RoB2). Two investigators (IHA and NMA) independently assessed each study, and no automation tools were used in this process^24^. We also employed the GRADE (Grading of Recommendations Assessment, Development, and Evaluation) criteria to provide a broader assessment of the confidence in the overall evidence supporting the outcomes of interest. We conducted publication bias analysis using funnel plots for primary outcomes using RevMan 5.4 software and considered statistical tests for funnel plot asymmetry, such as Egger’s test and Begg’s test.

### Data synthesis

We performed meta-analyses using Review Manager RevMan software (version 5.4). For continuous outcomes, we calculated standardized mean difference (SMD) or mean difference (MD) with 95% confidence interval (CI). For dichotomous outcomes, we used risk ratios (RR). We used the fixed effect model with homogenous data.

Heterogeneity was assessed using the I² statistic and p-value of heterogeneity, with p<0.1 indicating high heterogeneity. The thresholds for the I² statistic were as follows: 0–40% might not be important, 30–60% may represent moderate heterogeneity, 50–90% may represent substantial heterogeneity, and 75–100% considerable heterogeneity^20^. When significant heterogeneity was detected (p<0.1), we used a random effect model and conducted sensitivity analyses by excluding studies that contributed significantly to the heterogeneity.

We decided which studies were eligible for each synthesis by tabulating the study’s primary and secondary outcomes and comparing them against the planned groups for each synthesis. Methods required to prepare the data for presentation or synthesis included handling missing summary statistics by contacting study authors for clarification or using imputation methods where appropriate and data conversions when necessary.

## RESULTS

**Figure 1:**
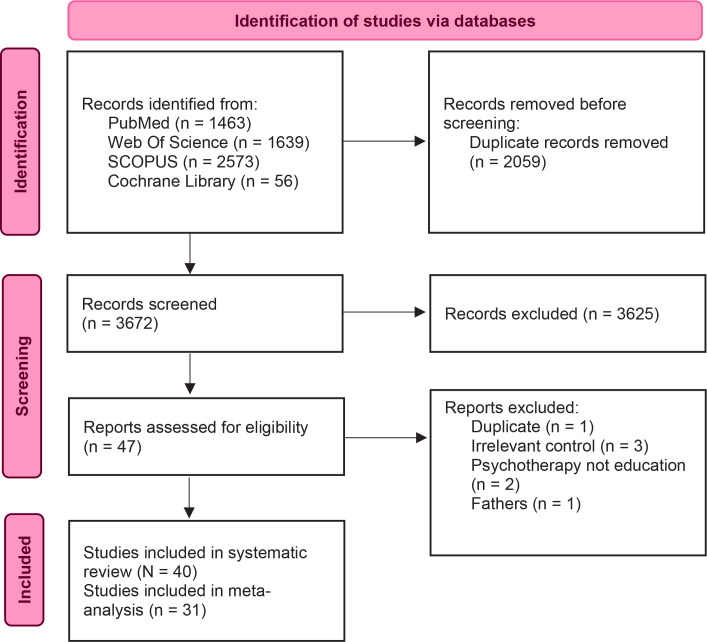
Flow diagram of study selection and inclusion process of the systematic review for the role of antenatal education on maternal self-efficacy, fear of childbirth, and birth outcomes

Our search yielded 5731 PubMed, Web of Science, Cochrane Library, and SCOPUS records. After removing 2059 duplicates, 3672 records underwent title and abstract screening, resulting in 3625 exclusions. The full-text screening was conducted on 47 reports, excluding seven additional records due to duplicates, having irrelevant controls, addressing psychotherapy instead of education, or focusing on fathers. Finally, 40 studies were included in the systematic review, with 31 eligible for meta-analysis (Figure 1).

### Baseline and characteristics of the included studies

The included studies were conducted in diverse settings, such as Turkey, Iran, Jordan, Nigeria, Denmark, and the USA. The educational content of antenatal courses varied widely, covering reproductive system changes, pregnancy physiology, birth preparedness, postpartum care, and breastfeeding techniques. The study duration ranged from a few months to several years. Participants’ gestational weeks at the start of interventions ranged from as early as 12 weeks^25^ to the last trimester. Sample sizes ranged from 29 to 13737 participants. The mean age of participants also varied widely, with studies reporting means ranging from 18.20 to 32.8 years (Table 1)^25-63^.

**Table 1:** Summary and baseline of the included studies

*Author Year*	*Location*	*Study duration*	*Educational content*	*Gestational weeks*	*Sessions*	*Primary outcomes*	*Follow-up*	*Conclusions*	*Study arms*	*Sample*	*Age (years) Mean ± SD*
Aba et al.^25^ 2017	Turkey	August 2011 and October 2013	Reproductive system, pregnancy physiology, maternal changes, exercise, postpartum care, breastfeeding	12–17	6	PSEQ, PPSEQ, NPI	The followup spanned approximately 30 weeks, from the 12th week of pregnancy to the fourth week after delivery	Antenatal education improved prenatal and early postpartum adaptation	AN	35	18.20 ± 0.99
									Control	35	18.03 ± 0.89
Abbasi et al.^26^ 2017	Iran	October 2015 and April 2016	Pregnancy modification, exercises, breathing, relaxation	32–36	Weekly reminders	CBSEI	Till delivery	E-learning and booklets boosted childbirth confidence	AN-Software	50	25.5 ± 3.8
									AN-Booklet	51	25.9 ± 3.6
									Control	52	25.1 ± 3.2
Abuidhail et al.^27^ 2019	Jordan	-	Breastfeeding benefits, techniques, problems, storage	29–36	2 weeks access	Breastfeeding knowledge, attitude, self-efficacy	Two weeks postpartum	No significant differences, but it may enhance selfefficacy	AN	59	27.7 ± 4.9
									Control	59	
Akinwaare et al.^28^ 2023	Nigeria	March 2019 and January 2020	Birth preparedness, complication readiness	20–24	20-min + 10-min interactive	BPCR, institutional delivery	Twelve weeks post-intervention	Enhanced birth preparedness and institutional delivery rates	AN	200	27.4 ± 4.9
									Control	200	27.1 ± 5.1
Aksoy Derya et al.^29^ 2021	Turkey	April and May, 2020	Tele-education on pregnancy, birth, COVID-19	Last trimester	5 (15–20 min each)	Prenatal distress, pregnancy anxiety	After one-week tele-education intervention	Reduced distress and anxiety	AN	48	28.70 ± 4.73
									Control	48	28.06 ± 4.12
AlSomali et al.^30^ 2019	Saudi Arabia	-	Antenatal education on pregnancy, birth, COVID-19	28–33	2 (2 h 50 min each)	Prenatal distress, pregnancy anxiety	Pretest and posttest data collection	Boosted maternal self-efficacy	AN	46	-
									Control	48	
Aslantekin Özçoban et al.^31^ 2022	Turkey	July 2018 to April 2019	Birth preparation	Second trimester	15 (3/week, 5 weeks)	Pregnancy acceptance, motherhood role, birth fear, selfefficacy, health literacy	Pretest and posttest data collection	Improved most scales except birth fear	AN	56	-
									Control	73	
Citak Bilgin et al.^32^ 2019	Turkey	September 2015 to June 2017	Delivery fear, birth stages, pain management, newborn care	Second trimester	5/weekly	POBS, BSES-SF, VAS	One month post-birth	Improved birth perception and breastfeeding selfefficacy	AN	65	27.54 ± 3.78
									Control	57	27.23 ± 4.82
Brixval et al.^33^ 2016	Denmark	August 2012 to May 2014	NEWBORN program	25–35	3 (2.5 h each)	Epidural use	Till 9 weeks postpartum	No difference in pain relief or interventions	AN	883	30.7 ± 4.1
									Control	883	30.8 ± 4.1
Calpbinici et al.^34^ 2022	Turkey	August 2019 to February 2020	Motivational interview for childbirth fear	24–28	-	W-DEQ A/B, CBSEI-SF	Till 24 hours after delivery	Reduced fear, increased selfefficacy, no impact on delivery mode	AN	37	-
									Control	36	
Çankaya et al.^24^ 2020	Turkey	April to September 2019	Birth fear, dynamics, coping, postpartum care	20–32	8 (4 h each)	Fear, self-efficacy, anxiety, stress, depression, delivery mode	6–8 weeks postpartum	Significant clinical benefits during pregnancy and postpartum	AN	57	26.4 ± 3.1
									Control	59	25.3 ± 3.7
Dai et al.^35^ 2021	China	October 2018 to November 2019	Simulationbased childbirth education	24–32	4 (70 min each)	WDEQ-A, CBSEI	Till delivery	An effective method for childbirth education	AN	26	28.42 ± 2.53
									Control	30	28.20 ± 2.19
Desmawati et al.^36^ 2019	Indonesia	June 2016 to January 2017	Non- pharmacological pain relief, Islamic praying	32	Daily until delivery	VAS, PBOS	Till delivery	Reduced pain, increased pain behaviors	AN	41	-
									Control	42	
Duncan et al.^37^ 2017	USA	In 2014	Mindfulnessbased childbirth and parenting	29–36	18-h weekend workshop	CBSEI	6 weeks postpartum	Improved childbirth appraisals, reduced postpartum depression risk	AN	15	-
									Control	14	
Escott et al.^38^ 2005	England	February to October 2000	Coping strategy enhancement vs standard	32	5 (2 h each)	Coping strategy use, pain, emotional experience	Three days pot delivery	Enhanced coping strategy use and birth companion involvement	AN	20	29 ± 5.9
									Control	21	29 ± 6.7
Firouzan et al.^39^ 2020	Iran	February to September 2019	BELIEF approach, telephone counseling	20–23	2 face-to-face, 8 phone	W-DEQ, childbirth self-efficacy, preference	Post-test assessments after the intervention	Reduced fear, increased selfefficacy	AN	35	26.27 ± 4.48
									Control	33	25.87 ± 4.58
Franzon et al.^40^ 2019	Brazil	August 2015 to February 2016	PRENACEL program	<20	4 texts/week	Preparedness, outcomes, intervention knowledge, care satisfaction	Until hospital discharge	Improved preparedness for birth	AN	116	-
									Control	440	
Gandomi et al.^41^ 2022	Iran	May to September 2017	Self-efficacy focused intervention	26–28	8 (90 min each)	Anxiety, neonatal outcomes	One month after the intervention	Reduced anxiety, improved pregnancy outcomes	AN	30	23.8 ± 3.31
									Control	30	23.3 ± 3.60
Gao et al.^42^ 2012	China	July 2008 to May 2009	IPT-oriented childbirth education	>28	2 (90 min) + phone follow-up	PSSS, PSOC-E, EPDS, GHQ	Three months postpartum	Beneficial for first-time Chinese mothers	AN	96	28.47 ± 2.80
									Control	98	28.38 ± 2.73
Hatamleh et al.^43^ 2019	Jordan	July to September 2016	Class-based program + WhatsApp	≥32	3 (40 min each)	Birth outcomes, breastfeeding initiation	48 hours post-birth	Increased spontaneous labor onset, earlier breastfeeding	AN	64	32.8 ± 3.65
									Control	64	
Hatamleh et al.^44^ 2023	Jordan	July to September 2016	Individualized childbirth education	≥32	3 (40 min each)	CBSEI, STAI	3 weeks after completing all educational sessions	Enhanced coping, reduced anxiety	AN	64	23.8 ± 3.91
									Control	64	
Ip et al.^45^ 2009	Hong Kong	August 2003 and April 2004	Self-efficacy and coping skills	32–34	2 (2 h 50 min each)	OE, EE, anxiety, pain, coping	24–48 hours after delivery	Promoted self-efficacy, reduced pain and anxiety	AN	60	27.88 ± 5.07
									Control	73	27.81 ± 5.09
Khademioore et al.^46^ 2023	Iran	February to April 2020	Tele-midwifery application	26–29	8 weeks, 3–4 msgs/day	FOC, self-efficacy, delivery mode	Two hours after birth	Reduced fear, increased self-efficacy, decreased C-sections	AN	35	24.3 ± 3.5
									Control	35	25.6 ± 3.5
Kronborg et al.^47^ 2012	Denmark	May 2006 to May 2007	‘Ready for Child’ program	30–35	3 (3 h each)	Breastfeeding duration	One year postpartum	Increased breastfeeding confidence and knowledge	AN	587	28.9 ± 3.7
									Control	575	29.2 ± 3.7
Madhavanprabha- karan et al.^48^ 2017	India	Between 2004 and 2005	Planned childbirth educational program	Third trimester	3	Anxiety, knowledge, pregnancy outcomes	Two weeks after the final educational session	Reduced anxiety and adverse outcomes	AN	50	-
									Control	50	
Maimburg et al.^49^ 2010	Denmark	May 2006 to May 2007	‘Ready for Child’ program	30–35	3 (3 h each)	Birth process, interventions, experience	One year postpartum	Improved coping with the birth process	AN	587	28.9 ± 3.7
									Control	575	29.2 ± 3.7
Maimburg et al.^50^ 2013	Denmark	May 2006 to May 2007	‘Ready for Child’ program	30–35	3 (3 h each)	Cambridge Worry Scale	One year postpartum	Lower worry levels, especially birth-related	AN	587	28.9 ± 3.7
									Control	575	29.2 ± 3.7
Mehdizadeh et al.^51^ 2005	Iran	July 2000 to March 2001	Birth preparation classes	>20	8	Pain, daily activity	Till delivery	Improved maternal and newborn health	AN	100	-
									Control	100	
Mohaghegh et al.^52^ 2023	Iran	December 2020 to June 2021	Childbirth preparation + birth plan	32–33	8 (90 min) + 1	Birth mode, labor duration, satisfaction	12–24 hours after birth	Increased normal births and satisfaction	AN	150	29.11 ± 4.72
									Control	150	28.90 ± 4.81
Mullany et al.^53^ 2006	Nepal	August 2003 to January 2004	Pregnancy care, birth preparedness	16–28	2 (35 min each)	Birth preparedness, healthcare utilization	Postpartum discharge	No significant differences in outcomes	AN	148	22.0 ± 3.6
									Control	149	22.6 ± 3.3
Noel-Weiss et al.^54^ 2006	Canada	August 2004 to February 2005	Prenatal breastfeeding workshop	>34	1 (2.5 h)	Breastfeeding self-efficacy, duration	8 weeks postpartum	Improved self-efficacy, increased exclusive breastfeeding	AN	47	-
									Control	45	
Öztürk et al.^55^ 2022	Turkey	November 2016 to January 2018	Breastfeeding education	Pre-delivery	2 (4 h each)	Breastfeeding self-efficacy, success	One week postpartum	Enhanced self-efficacy, increased success	AN	34	-
									Control	33	
Rahimparvar et al.^56^ 2012	Iran	October 2010 to February 2011	Educational software (CD)	28–32	Accessible anytime	CBSEI, STAI	Till delivery	Improved self-efficacy in coping with labor	AN	75	25.17 ± 3.89
									Control	75	24.79 ± 4.21
Sabri Piro et al.^57^ 2020	Iraq	October 2017 to July 2018	Breastfeeding education	30–38	2 (60–90 min each)	BF knowledge, attitudes, self-efficacy	Two months after childbirth	Increased self-efficacy, promoted exclusive BF	AN	65	26.80 ± 6.60
									Control	65	26.38 ± 6.80
Serçekuş et al.^58^ 2016	Turkey	March 2012 to January 2014	Comprehensive antenatal education	26–28	8 (120 min each)	W-DEQ, CBSEI, MAI, PPAQ	6 months postpartum	Recommended implementation in developing countries	AN	31	28.8 ± 2.2
									Control	32	27.7 ± 4.5
Turkstra et al.^59^ 2017	Australia	May 2012 to June 2013	Telephone psychoeducation	24–34	2	WDEQ-A	6 months postpartum	No enhancement in health-related quality of life	AN	95	30.5 ± 4.98
									Control	89	30.2 ± 5.82
Uludağ et al.^60^ 2022	Turkey	October to December 2021		24–34	8 (4 h each)	OWLS, FOBS, PSEQ, FCV-19S	Post-test assessments after the intervention	Improved labor preparedness during COVID-19	AN	23	26.69 ± 4.93
									Control	21	25.66 ± 4.58
Xie et al.^61^ 2018	China	October 2011 to August 2012	WHO materials via text messages	Throughout	Text messages	Maternal/perinatal outcomes	Within 42 days postpartum	No significant differences	AN	6771	-
									Control	6966	
Yesildag et al.^62^ 2024	Turkey	January to July 2022	Web-based program + motivational interviews	28–30	5 weeks	W-DEQ A, CSES, BHBS	Till 24 hours after delivery	Reduced fear, increased self-efficacy	AN	37	-
									Control	36	
Zafman et al.^63^ 2023	USA	April 2021 to April 2022	Birthing online program	<20	5 courses	PrAS	Postpartum discharge	Reduced anxiety, decreased emergency care use	AN	45	23.7 ± 4.5
									Control	45	23.7 ± 4.7

AN: antenatal. CBSEI: Childbirth Self-Efficacy Inventory. PSEQ: Prenatal Self-Efficacy Questionnaire. PPSEQ: Postpartum Self-Efficacy Questionnaire. NPI: Neonatal Perception Inventory. BPCR: Birth Preparedness and Complication Readiness. CBSEI-SF: Childbirth Self-Efficacy Inventory-Short Form. W-DEQ A/B: Wijma Delivery Expectancy/Experience Questionnaire A/B. POBS: Pain Outcomes Brief Scale. BSES-SF: Breastfeeding Self-Efficacy Scale-Short Form. VAS: Visual Analog Scale. NEWBORN: Newborn Care Program. PSSS: Perceived Social Support Scale. PSOC-E: Parent Stress Index-Early Childhood. EPDS: Edinburgh Postnatal Depression Scale. GHQ: General Health Questionnaire. FOC: fear of childbirth. OE: overall experience. EE: emotional experience. CSES: Coping Self-Efficacy Scale. BHBS: Breastfeeding and Health Behavior Scale. OWLS: Overall Wellness Scale. FOBS: Fear of Birth Scale. FCV-19S: Fear of COVID-19 Scale. PrAS: Pregnancy and Childbirth Anxiety Scale.

### Quality assessment

Four studies were classified as having a high risk of bias using the RoB2 quality assessment tool, three of which showed high risk in the domain of missing outcome data and one study in the randomization process. Another five studies were classified as having some concerns, with four showing some concerns in the domain of the randomization process and one study in the selection of the reported result domain. The other 31 were rated as having an overall low risk of bias (Supplementary file Figures 1 and 2).

### Primary outcomes

*Change in self-efficacy on childbirth*

**Figure 2:**
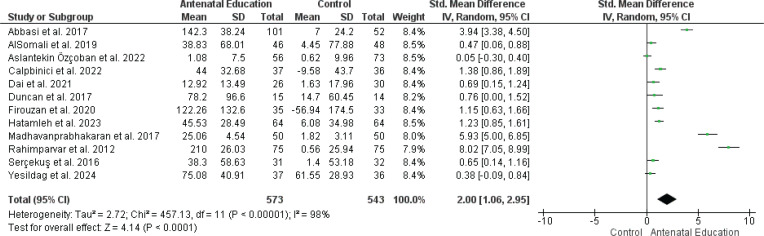
Forest plot of the effect of antenatal education on childbirth self-efficacy

The pooled analysis of 12 RCTs, involving 1116 pregnant women, showed a significant increase in the self-efficacy on childbirth in the group that received antenatal (AN) education compared with the control group (SMD=2.00; 95% CI: 1.06–2.95, p<0.0001) (Figure 2). The data showed unresolvable heterogeneity (p<0.00001, I^2^=98%), which could not be adequately explained or reduced even after conducting additional analyses (e.g. subgroup analyses, sensitivity analyses, or using different statistical models). Possible causes of heterogeneity among study results include variations in study design, population characteristics, and intervention protocols. There appears to be some evidence of publication bias for self-efficacy, as the funnel plot shows an asymmetrical distribution, suggesting a potential bias (Supplementary file Figure 3).

*Change in fear of childbirth*

**Figure 3:**
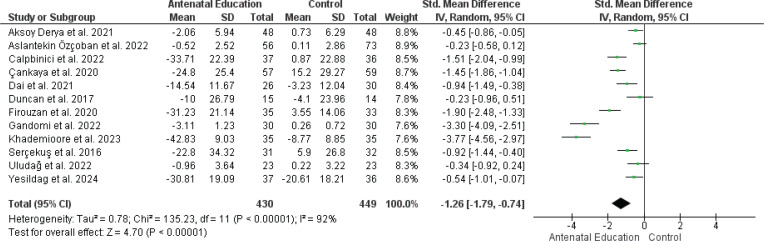
Forest plot of the effect of antenatal education on fear of childbirth

AN education significantly decreased the fear of childbirth in pregnant women compared with the control group based on the pooled analysis of 12 RCTs, including 879 women (SMD= -1.26; 95% CI: -1.79 – -0.74, p<0.00001) (Figure 3). Still, the data showed unresolvable heterogeneity (p<0.00001, I^2^=92%), which could not be adequately explained or reduced after additional analyses. Possible causes of heterogeneity include differences in the measurement tools used and the timing of the interventions. The fear of birth funnel plot also shows some asymmetry, indicating a possible bias (Supplementary file Figure 4).

### Secondary outcomes

*Maternal outcomes*

Frequency of vaginal delivery

**Figure 4:**
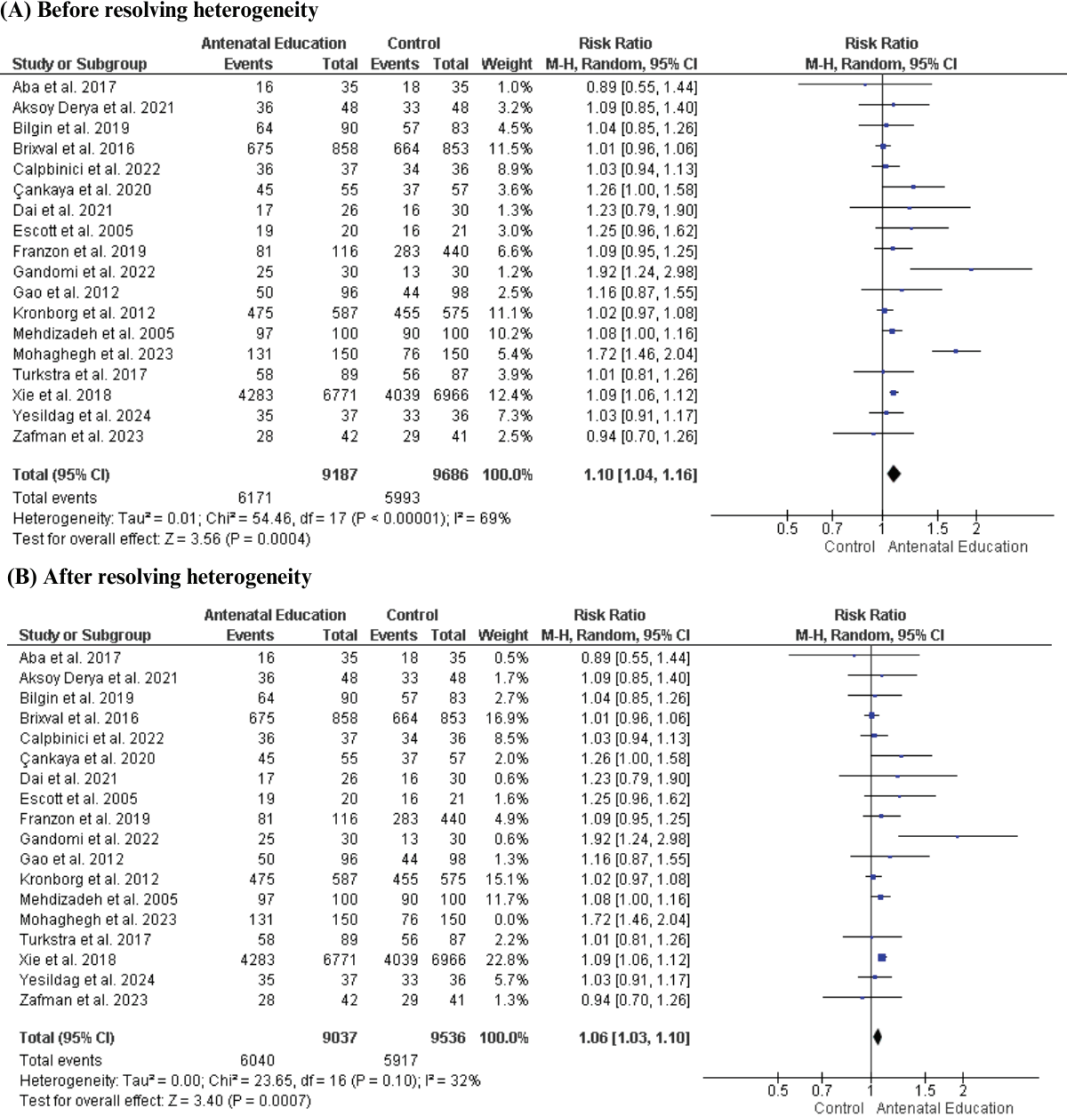
Forest plot of the effect of antenatal education on rate of vaginal delivery: A) Before resolving heterogeneity; B) After resolving heterogeneity

The rate of vaginal delivery showed a significant increase among women who received AN education compared with the control group based on the pooled analysis of 18 RCTs involving 18873 women (RR=1.10; 95% CI: 1.04– 1.16, p=0.0004); however, the data were heterogeneous (p<0.00001, I^2^=69%). This heterogeneity was resolved by excluding the study of Mohaghegh et al.^52^ (p=0.10, I^2^=32%), and the results remained significant (RR=1.06; 95% CI: 1.03–1.10, p=0.0007) (Figure 4).

Frequency of cesarean section

**Figure 5:**
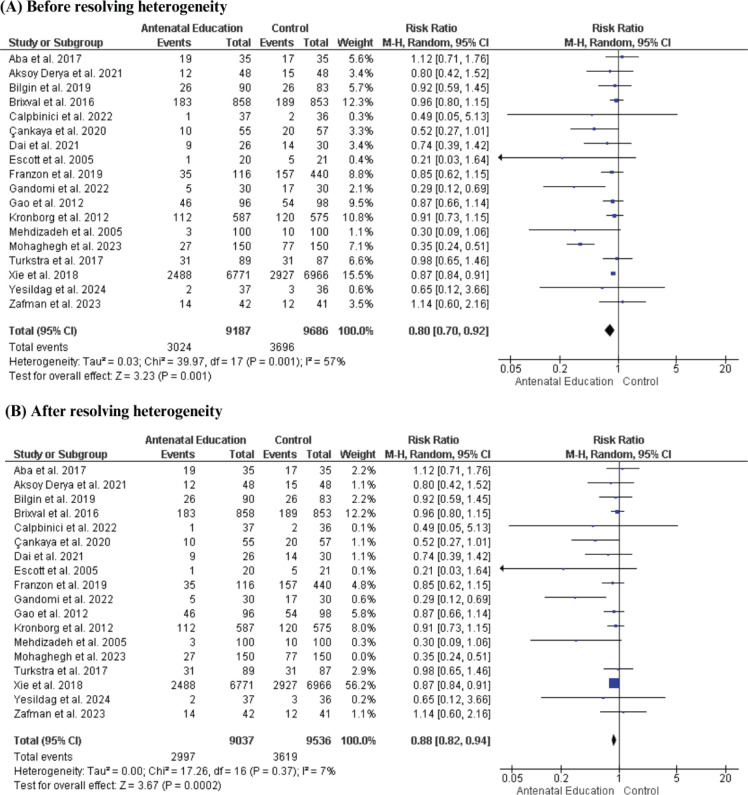
Forest plot of the effect of antenatal education on rate of cesarean section: A) Before resolving heterogeneity; B) After resolving heterogeneity

In contrast, the pooled analysis of 18 RCTs, including 18873 women, revealed a significantly lower cesarean section rate in the AN education group compared with the control group (RR=0.80; 95% CI: 0.70–0.92, p=0.001). Still, the data showed heterogeneity (p=0.001, I^2^=57%). This heterogeneity was resolved by excluding the study of Mohaghegh et al.^52^ (p=0.37, I^2^=7%), and the results remained significant (RR=0.88; 95% CI: 0.82–0.94, p=0.0002) (Figure 5).

Frequency of episiotomy

There was an insignificant difference between the two groups in the episiotomy rate (RR=1.16; 95% CI: 1.00–1.34, p=0.06), and the data were homogenous (p=0.71, I^2^=0%) (Supplementary file Figure 5).

*Neonatal outcomes*

Apgar score after one and five minutes

There was an insignificant difference between the AN education and the control group of neonatal APGAR score after one and five minutes (MD=0.05; 95% CI: -0.04–0.14, p=0.24) and (MD=0.03; -0.03–0.10, p=0.30), respectively. The data were homogenous in both analyses (p=0.72, I²=0%) and (p=0.73, I²=0%) (Supplementary file Figure 6).

Infant’s birth weight

There was an insignificant difference between the two groups in the infant weight (MD=19.13; 95% CI: -81.23–119.48, p=0.71), but the data were heterogeneous (p=0.0006, I²=77%). Even after resolving heterogeneity by excluding Citak Bilgin et al.^32^ (p=0.14, I²=42%), the results remained insignificant (MD= -36.12; 95% CI: -99.16–26.92, p=0.26) (Supplementary file Figure 7).

Incidence of low birth weight (<2500 g)

Finally, there was an insignificant difference between AN education and control groups in the incidence of low birth weight (RR=0.98; 95% CI: 0.83–1.17, p=0.85), and the data were homogenous (Supplementary file Figure 8).

### GRADE assessment

According to GRADE, all our comparisons in the different outcomes were at varying levels of certainty (from low to moderate) (Supplementary file Table 2).

## DISCUSSION

Our meta-analysis reveals that antenatal (AN) education programs significantly positively impact key psychological and clinical outcomes for expectant mothers. The primary results demonstrate an increase in childbirth self-efficacy and a notable decrease in fear of childbirth among women who participated in AN education compared to control groups. Secondary outcomes show improvements in maternal outcomes, such as increased rates of vaginal delivery and decreased rates of cesarean sections. These findings suggest that such programs effectively empower women with knowledge and confidence, potentially enhancing their ability to cope with the challenges of childbirth. The novelty of this meta-analysis lies in its comprehensive evaluation of both psychological and clinical outcomes, providing robust evidence for the multifaceted benefits of AN education programs.

The observed improvements in self-efficacy and reduced fear may be attributed to several factors inherent in antenatal education programs^23^. These programs typically provide comprehensive information about pregnancy, labor, and delivery, which can demystify the process and alleviate anxiety stemming from the unknown^6^. Additionally, many antenatal classes incorporate practical coping strategies and relaxation techniques, equipping women with tangible skills to manage pain and stress during childbirth^64^. The group setting of many programs may also foster a sense of community and shared experience, further bolstering confidence and reducing isolation-related fears^65^.

Our analysis revealed significant clinical benefits associated with AN education. Women who received AN education showed higher rates of vaginal delivery and lower rates of cesarean section. These outcomes may be directly linked to the psychological benefits observed. Increased self-efficacy and reduced fear could contribute to more relaxed and confident mothers, potentially facilitating smoother labor progression and reducing the likelihood of interventions^66^. Moreover, educated mothers may be better equipped to make informed decisions about their care, possibly leading to fewer unnecessary cesarean sections^67^.

Interestingly, we found no significant differences in episiotomy rates, Apgar scores, infant birth weight, or incidence of low birth weight between the intervention and control groups. This suggests that while AN education has clear benefits for maternal psychological well-being and mode of delivery, its impact on specific obstetric and neonatal outcomes may be limited. These results highlight the complex interplay of factors influencing childbirth outcomes and underscore the need for comprehensive prenatal care beyond education alone.

Our results align with those of Zanetti et al.^8^. Our meta-analysis demonstrates a significant increase in childbirth self-efficacy (SMD=2.00; 95% CI: 1.06–2.95, p<0.0001). Zanetti et al.^[Bibr CIT0008]8^ also observed significant improvements, reporting outcome expectancy scores of 16.00 and efficacy expectancy scores of 20.44. Both studies highlight the positive impact of AN education on self-efficacy, with our standardized mean difference offering a more generalizable effect size. We found a significant increase in vaginal delivery rates (RR=1.10; 95% CI: 1.04–1.16, p=0.0004), consistent with the Zanetti et al.^8^ findings of increased frequency (OR=1.28). Both studies indicate that AN education is associated with higher rates of vaginal delivery, with our larger sample size yielding a more precise estimate. We found no significant difference in episiotomy rates (RR=1.16; 95% CI: 1.00–1.34, p=0.06), aligning with the Zanetti et al.^8^ results.

Our study explored additional outcomes not covered by Zanetti et al.^8^ including fear of childbirth, cesarean section rates, and various neonatal outcomes. This comprehensive approach provides a broader perspective on the effects of antenatal education. Our meta-analysis, encompassing 40 studies in the systematic review and 31 in the meta-analysis, offers potentially more robust and generalizable conclusions than the Zanetti et al.^8^ analysis of nine studies.

Nevertheless, our findings on the fear of childbirth are consistent with Stoll et al.^68^, who reported that psycho-education interventions effectively reduce the fear of childbirth. Our study further corroborates this by focusing on antenatal education programs and their impact on fear and self-efficacy. Meanwhile, our results indicating increased vaginal delivery rates and decreased cesarean section rates align with the Cochrane review by Sandall et al.^69^, which found that midwife-led continuity models of care, often incorporating comprehensive antenatal education, are associated with higher rates of spontaneous vaginal birth.

The consistent positive effects of AN education on maternal psychological outcomes and mode of delivery have significant implications for maternity care practices. These findings suggest that investing in comprehensive and accessible AN education programs could be a cost-effective strategy to enhance maternal experiences and clinical outcomes. By boosting self-efficacy and reducing fear, these programs may lead to more positive birth experiences, potentially lowering the risk of postpartum depression and improving mother-infant bonding. However, the lack of significant impact on certain neonatal outcomes indicates that while AN education is beneficial, it should be part of a broader prenatal care approach. This approach may include addressing social determinants of health, ensuring adequate nutrition, and providing comprehensive medical care throughout pregnancy. From a policy perspective, these results support the integration of high-quality AN education as a standard component of maternity care. Healthcare systems and providers should prioritize developing and implementing evidence-based education programs that are culturally appropriate and accessible to diverse populations.

### Strengths and limitations

A key strength of our study is its comprehensive nature, including many randomized controlled trials (RCTs) and a substantial combined sample size. This provides strong evidence for the effectiveness of antenatal education. Additionally, our analysis considered both psychological and clinical outcomes, offering a comprehensive view of the impacts of these programs. Another strength of our study is that we exclusively included RCTs as part of the inclusion criteria for study selection.

However, several limitations should be noted. The potential risk of bias in the included studies and the high heterogeneity observed in some analyses suggest considerable intervention and outcome variability across studies. While partly resolved through sensitivity analyses, this heterogeneity indicates the need for caution in interpreting and generalizing results. Furthermore, the potential publication bias identified for primary outcomes suggests that positive results may be overrepresented in the literature. Another limitation is the variability in antenatal education programs’ content, duration, and delivery methods across studies. Publication bias in this meta-analysis may arise from several factors, including the tendency to publish studies with positive findings, selective outcomes reporting, and excluding studies not in English. Despite these biases, the results of our meta-analysis can still be generalized to a broader population due to the large sample size and the inclusion of diverse study settings. However, caution should be exercised when interpreting the findings, and further research is needed to confirm these results in different contexts and populations. This makes it challenging to identify specific components that are most effective. Most of the studies included were conducted in middle- to high-income countries, which could restrict the applicability of the findings to low-resource settings.

## CONCLUSIONS

Our meta-analysis provides strong evidence for the benefits of antenatal education in improving maternal psychological outcomes and promoting vaginal delivery. These findings underscore the importance of integrating high-quality antenatal education into routine prenatal care. Future research should focus on identifying the most effective components of these programs and exploring their long-term impacts on maternal and child health. Additionally, efforts should be made to develop and evaluate culturally adapted antenatal education interventions for diverse populations, particularly in low-resource settings. Policymakers and healthcare providers should prioritize the implementation and accessibility of evidence-based antenatal education programs as a key strategy to enhance maternal and neonatal outcomes.

## Data Availability

Data sharing is not applicable to this article as no new data were created.
